# New “HOPE” laser for photoacoustic imaging of water

**DOI:** 10.1038/s41377-022-00805-9

**Published:** 2022-04-26

**Authors:** Ji-Xin Cheng

**Affiliations:** Boston University Photonics Center, Boston, MA 02215 USA

**Keywords:** Imaging and sensing, Optical sensors

## Abstract

A hybrid optical parametrically-oscillating laser at 1930 nm enables photoacoustic mapping of water content in deep tissue with good sensitivity and high spatial resolution.

Water is of vital important to life. Tracing the water distribution with high precision is essential to study the metabolism of a living system. Back to almost 100 years ago, in 1933, the distribution and the dynamics of water inside the human body was studied^1^. With the fast development of advanced laser and electrical techniques, significant advances have been made to sense the water in the biological system. The spatial distribution of the intracellular hydrogen bonding was mapped with high sensitivity and resolution using confocal Raman microscopy^2^, stimulated Raman excited fluorescence microscopy^3^, and fluorescence lifetime microscopy^4^. The map of the water content in the superficial area of the skin was visualized by coherent Raman scattering microcopy^5,6^. These powerful techniques successfully revealed the water heterogeneity in the biological system. However, the intrinsic optical scattering in the complex biological samples limited the penetration depth that these techniques can reach. Photoacoustic microscopy is a good candidate to image endogenous molecules with high depth-to-resolution ratio^7,8^. Photoacoustic microscopic imaging of water in the deep tissue essentially requires a high-power laser source of which the wavelength locates at the vibrational absorption peak of O–H bond^9^. In Fig. 1, the solid red curve indicates the absorption spectrum of water, which is proportional to the PA signal amplitude. The solid black curve combining the tissue scattering (as indicated in the solid gray curve) and the water absorption spectrum indicates the effective attenuation coefficient^10,11^, determining the penetration depth of PA imaging of water. Via computing the ratio between the water absorption and effective attenuation spectrum, it is seen that the peak is located at around 1.9 µm, indicating a 1.9-µm laser can serve to generate strong PA signal of water while keeping a large penetration depth. In this issue, the researchers from The University of Hong Kong and Technion—Israel Institute of Technology recently reported a novel hybrid optical parametrically-oscillating laser at 1930 nm and integrated it to a photoacoustic microscope. The authors successfully imaged the water content with deep penetration depth and high spatial resolution^12^. Their work opens a new road to visualize the water distribution in the biological system, facilitating more insights into the preclinical research and clinical applications.

**Fig. 1:**
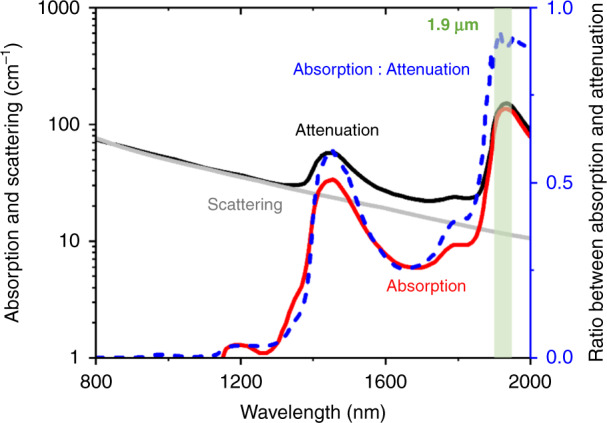
The absorption and scattering of water in the spectral window of 800–2000 nm^10,11^. The attenuation is a sum of absorption and scattering. The ratio of absorption to attenuation is peaked around 1900 nm

The novel 1930-nm laser source demonstrated in this work^12^ is a type of high-power all-fiber hybrid optical parametrically-oscillating emitter (HOPE). HOPE can emit high power 1930-nm laser with short pulse duration and fast repetition rate. Therefore, HOPE was suitably designed for the high-sensitivity and ultrafast photoacoustic imaging of water in the deep tissue. Here, HOPE was registered into a transmission-mode optical-resolution photoacoustic microscope (OR-PAM). The penetration depth demonstrated with the aqueous sample reached to 2.4 mm, revealing the capability of this technique to map the water distribution in deep tissue. The authors demonstrated high-sensitivity photoacoustic imaging of water in the adipose tissue in vitro with good signal-to-noise ratio and suppressed lipid signals. As such, it implies the good fidelity of this technique to image water in a complex biological environment.

Due to the strong vibration absorption of O–H bond and less photon scattering at 1930 nm, this 1930-nm OR-PAM demonstrated good sensitivity and deep penetration depth. The technique reported in this work will open a new road for photoacoustic imaging of water in relevant biological studies and clinical applications. For instance, via optical sensing method to detect the PA signals^13,14^, it is likely to achieve non-contract PA imaging of water in the deep tissue, facilitating this technique for more biological research or clinical applications on human body or small animals.

Overall, this paper reported a novel high-power, ultrafast 1930-nm laser, and it was registered with optical-resolution photoacoustic microscopy. This methodology provides a label-free imaging tool to visualize the water distribution with improved depth-to-resolution ratio. The 1930-nm OR-PAM achieves a good balance between sensitivity, penetration depth, spatial resolution, and minimal artefacts for imaging water. This advancement in optical imaging of water opens up many applications that require deep penetration depth, high resolution, and good imaging fidelity, such as cancer diagnosis and retinal imaging.
